# Physical activity and sedentary behavior following pediatric burns – a preliminary investigation using objective activity monitoring

**DOI:** 10.1186/s13102-018-0093-5

**Published:** 2018-02-09

**Authors:** Moniek Akkerman, Leonora J. Mouton, Laurien M. Disseldorp, Anuschka S. Niemeijer, Marco van Brussel, Lucas H. V. van der Woude, Marianne K. Nieuwenhuis

**Affiliations:** 1 Association of Dutch Burn Centres, Burn Centre Martini Hospital, Groningen, The Netherlands; 2 University of Groningen, University Medical Center Groningen, Center for Human Movement Sciences, Groningen, The Netherlands; 3 Martini Academy, Martini Hospital, Groningen, The Netherlands; 4 Child Development & Exercise Center, Wilhelmina Children’s Hospital, University Medical Center Utrecht, Utrecht, The Netherlands; 5 University of Groningen, University Medical Center Groningen, Center for Rehabilitation, Groningen, The Netherlands

**Keywords:** Exercise, Outcome assessment, Accelerometry, Rehabilitation, Burns

## Abstract

**Background:**

Adequate levels of regular physical activity (PA) are crucial for health and well-being. Pediatric burn injuries can have major physiological consequences in both the short and long term. The question is whether these consequences affect post burn PA levels. This study therefore aimed to describe PA and sedentary behavior (SB) in children and adolescents 1–5 years after burn injury.

**Methods:**

Daily PA and SB were monitored in 20 children and adolescents (12 boys and 8 girls, aged 6–17 years, with burns covering 10–37% of total body surface area, 1–5 years post burn) for 1 week using the ActiGraph GTX3+ accelerometer. Activity counts were categorized into SB, light PA, moderate PA, vigorous PA, moderate-to-vigorous PA (MVPA), and total PA. Outcomes were compared with non-burned reference values and PA levels recommended by the World Health Organization (WHO).

**Results:**

The participants spent about 5.1 h per day on total PA and 7.4 h on SB. Most of the active time (~ 83%) was categorized as light PA. Thirty-five percent of the group, especially the young boys, spent on average ≥ 60 min on MVPA per day. The boys, although with large interindividual differences, spent more time on MVPA than the girls (*p* < .005). Older age was associated with less PA time, while more time was spent sedentary. No trends were found indicating an effect of burn characteristics, time post burn, or length of hospital stay, and no differences were found with non-burned peers.

**Conclusion:**

Duration and intensity of PA and SB in children and adolescents 1–5 years after burn injury were similar to non-burned peers. However, only 35% of the group met the WHO physical activity recommendation. Given the increased long term risk for physical conditions following pediatric burns, physical activity should be encouraged in this vulnerable population.

**Trial registration:**

The study is registered in the National Academic Research and Collaborations Information System of the Netherlands (OND1348800).

**Electronic supplementary material:**

The online version of this article (10.1186/s13102-018-0093-5) contains supplementary material, which is available to authorized users.

## Background

Physical activity in children and adolescents is a widely publicized topic due to the increasing awareness of its significance for health and well-being [1]. Adequate levels of regular physical activity can improve muscular strength and cardiopulmonary endurance, help to prevent a number of chronic diseases throughout life, and are also essential for the social, emotional, and cognitive development of children and adolescents [1, 2]. In contrast, sustained sedentary behavior has been associated with negative health outcomes like cardiovascular diseases, type 2 diabetes, and metabolic syndrome [3].

Children with physical disabilities or chronic diseases tend to be more restricted in performing physical activity than their healthy peers [4]. This might also apply for children who have been hospitalized with burns, as a burn injury can have major physical and physiological consequences in both the short and long term that are expected to affect the time spent on physical activity and to encourage sedentary behavior. First, burns are associated with substantial loss of skeletal muscle mass and strength, due to amino acid depletion from the muscles for the formation of new skin [5], and prolonged periods of bed rest and immobilization. Secondly, burns covering > 30% of total body surface area can lead to hypermetabolism, which is frequently associated with cachexia [6]. In addition, there is preliminary evidence that burns can alter muscle energy metabolism, leading to an earlier onset of muscle fatigue and longer recovery periods following exercise [7]. In children and adolescents with severe burns, these burn induced metabolic and inflammatory changes have been shown to persist for 3 years after the injury [6]. Following less extensive burns, it is yet unknown whether pathophysiological alterations affect physical functioning after 1 year. Besides these physiological issues, it is feasible that children with burns experience additional barriers to physical activity, like fatigue [8], anxiety, pain, limited flexibility due to scar contractures, or psychosocial problems like difficulties with accepting their altered appearance [9].

Recently evidence emerged about long term physical health outcomes in the pediatric burn population [10–14]. A long term follow-up study showed that burn injured children had an increased risk of arthritis, fractures, and pulmonary conditions compared to their non-burned peers, even following non-severe burns [10]. Accordingly, pediatric burn patients had increased hospital admission rates for respiratory infection [11], cardiovascular diseases [12], and musculoskeletal diseases [13], and even an increased risk of mortality in the long-term [14]. Although the specific causes of those physical and physiological consequences years beyond the burn injury have yet to be identified, lack of physical activity might play a role.

To identify whether health and well-being of children and adolescents after burn injury are at risk due too inadequate daily physical activity and/or too much sedentary behavior, it is important to become aware of their daily time spent in both types of behavior. Although physical fitness after pediatric burns received more attention during the last decades [15–17], habitual physical activity (which is intrinsically associated with physical fitness) has not been assessed before. Therefore, the current study aimed to describe daily time spent in various intensities of physical activity and sedentary behavior in children and adolescents with a wide range of burn characteristics, using objective activity monitoring, and to compare these results with non-burned reference values.

## Methods

The data described in this study were obtained as part of a cross-sectional descriptive study, performed by our study group, regarding physical activity and fitness following pediatric burns [18]. The entire study involved not only the assessment of physical activity and sedentary behavior, but also assessment of physical fitness, fatigue, and health-related quality of life. Study procedures were described previously in detail [18].

### Study population

Eligibility criteria were 6–18 years of age, involvement in a burn accident 0.5–5 years ago, admission to one of the three Dutch Burn Centers with burns covering > 10% of their total body surface area and/or a length of hospital stay of more than 6 weeks. The national Dutch Burn Repository was used to identify potentially eligible patients. Children with extensive (pre-existing) comorbidity, insufficient Dutch language proficiency, or (mental) disabilities were excluded. In case participants had reconstructive surgery less than 2 months before the time of planned assessment, the assessment was postponed.

### Accelerometry: Data collection and analysis

Daily physical activity and sedentary behavior were monitored using the triaxial ActiGraph GTX3+ accelerometer (ActiGraph, Pensacola, Florida, U.S.A.). This wearable activity monitor converts acceleration signals into samples that are summed over a user-specified time sampling interval, called *epoch*. At the end of each epoch, the summed value is stored in the monitor memory as activity counts. The Actigraph GT3X+ has been shown to be a valid and reliable instrument to assess frequency, intensity, and duration of physical activity and sedentary behavior in children and adolescents [19].

**Fig. 1:**
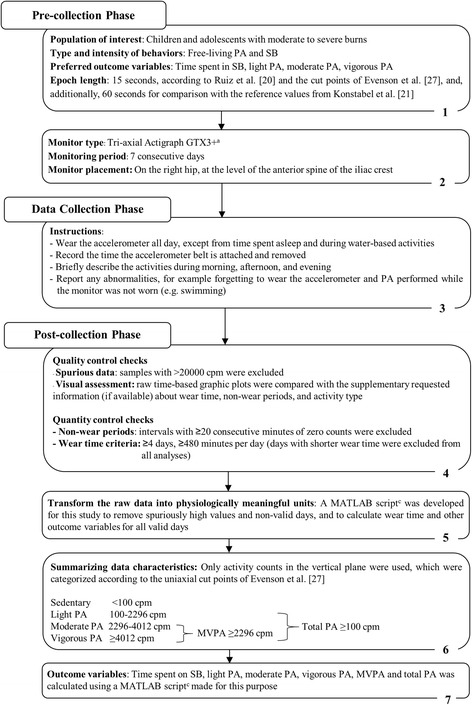
Process of handling the accelerometer data according to the recommended 7-step algorithm of Heil et al. [22]. Abbreviations: PA = physical activity, SB = sedentary behavior, cpm = counts per minute, MVPA = moderate-to-vigorous physical activity

Monitors were initialized using the Actilife software (Actilife software, version 6.7.3, ActiGraph, Pensacola, Florida, U.S.A.) to collect activity counts at 100 Hz. To enable comparison with European non-burned reference values [20, 21], only accelerometer measurements in the vertical plane were used. The previously published non-burned reference groups consisted of 4936 European children (2411 boys and 2525 girls, aged 6–11 years) [21] and 2200 European adolescents (1016 boys and 1184 girls, aged 12.5–17.5 years) [20]. Reference values were available for light physical activity, moderate-to-vigorous physical activity and sedentary behavior in children [21], and for moderate-to-vigorous physical activity and sedentary behavior in adolescents [20]. For optimal transparency of our data handling methods, the 7-step algorithm of Heil et al. [22] was used for collecting, processing, and summarizing the accelerometer data into physical activity and sedentary behavior outcome variables, according to best practice (Fig. 1). To be able to compare our activity data to those of the reference studies, the measurements needed to be comparable. Therefore, we chose to adopt the decisions in data reduction and analysis (epoch length, wear time criteria, non-wear time criteria, spurious data, cutpoints) from our reference studies. Data reduction and analysis were performed using MATLAB software (release 2015a, The MathWorks, Inc., Natick, Massachusetts, U.S.A.).

### Statistical analyses

Subject characteristics and descriptors of physical activity and sedentary behavior were presented as mean, standard deviation, and range, for boys and girls separately. Influence of sex was assessed using independent t-tests. To identify potential predictors of total physical activity, moderate-to-vigorous physical activity and sedentary behavior, exploratory multiple regression analyses (hierarchical, blockwise entry) were performed. As both age and sex are known predictors of physical activity and sedentary behavior [20, 21], these variables were entered simultaneously into the model first. Subsequently, burn characteristics (%total body surface area burned, full thickness burns yes(1)/no(0), legs involved yes(1)/no(0)), time post burn (years), and length of hospital stay (days) were entered separately, one by one, in order to identify whether the regression model could be improved by one or more of these potential predictors. For each regression model, the standard error of the estimate (SEE) was provided as an indication for the accuracy of the prediction by the model. The smaller the SEE, the more accurate the prediction.

To assess achievement of physical activity levels as recommended by the World Health Organization (WHO) (Table 1), average daily time spent in moderate-to-vigorous physical activity was calculated for each subject, and results were plotted together with a line representing the recommended daily minimum of 60 min.

**Table 1:** Outline of international definitions and recommendations of physical activity and sedentary behavior in children and adolescents

	Definition	Recommendations
Physical activity	Any bodily movement produced by skeletal muscles that requires energy expenditure – including activities undertaken while working, playing, carrying out household chores, travelling, and engaging in recreational pursuits [25].	Children and adolescents aged 5–17 should accumulate at least 60 min of moderate-to-vigorous physical activity daily. Most of the daily physical activity should be aerobic. Vigorous activities should be incorporated, including those that strengthen muscle and bone, at least 3 times per week [25].
Sedentary behavior	Any waking behavior, characterized by an energy expenditure ≤1.5 metabolic equivalents (MET’s), while in a sitting or reclining posture [47].	Children (aged 5–11 years) and adolescents (aged 12–17 years) should minimize the time they spend being sedentary each day. To achieve this:
		- Limit use of electronic media for entertainment (e.g. television, seated electronic games and computer use) to no more than 2 h per day [48, 49]
		- Limit sedentary (motorized) transport, extended sitting time, and time spent indoors throughout the day [48]
		- Break up long periods of sitting as often as possible [49]

For comparison with non-burned reference values, individual data were plotted together with age- and sex-matched European reference values (mean ± 2 SD). Subjects that deviated more than two standard deviations from the non-burned mean were assumed different from their non-burned peers.

IBM SPSS Statistics for Windows (version 20.0, IBM Corp, Armonk, New York, U.S.A.) was used for the statistical analyses. A two-sided *p*-value <.05 was considered statistically significant.

## Results

**Fig. 2:**
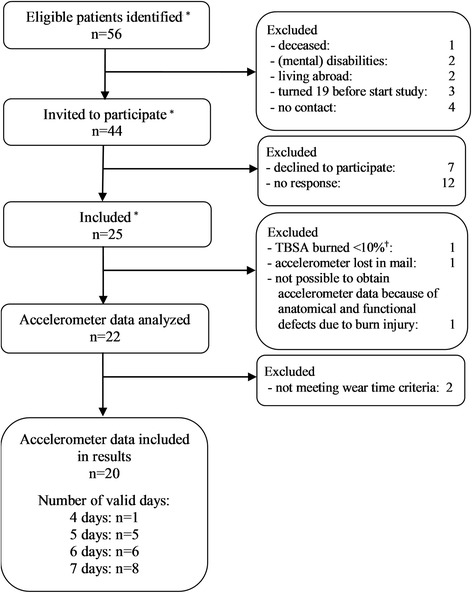
Flow of patients. ^*^ Study of Disseldorp et al. [17]. ^†^ This child was registered as having burns covering > 15% of total body surface area (TBSA). It emerged however that its burns had in fact affected < 5% of TBSA. Therefore, this child was excluded

Data from 20 children and adolescents with burns (12 boys and 8 girls, aged 6–17 years, with burns covering 10–37% of total body surface area) were included in the current study (Fig. 2, Table 2). Inhalation injury was not present in our study population and none of the burns were caused by chemical substances or electricity. No significant differences in subject characteristics were found between boys and girls (Table 2).

**Table 2:** Characteristics of the study population

Parameter	Boys (*n* = 12)			Girls (*n* = 8)			*p*-value^b^
	Mean	SD	Range	Mean	SD	Range	(Boys vs Girls)
Age (years)	10.8	3.7	6–17	9.6	2.7	6–14	.4424
%TBSA burned	18.3	8.4	10–37	13.5	2.8	10–17	.1391
full thickness burns	(8/12)			(5/8)			
lower extremity involved	(11/12)			(5/8)			
inhalation injury	(0/12)			(0/8)			
Surgeries (#)^a^	1		0–7	1		0–2	
Time post burn (years)	3.0	1.4	1–5	3.0	0.7	1–4	> .99
Length of stay (days)	25.8	7.9	18–42	27.3	11.8	16–55	.7362

Abbreviations: *%TBSA* percentage of total body surface area, *#* number, ^a^mode instead of mean ± SD. ^**b**^ independent sample *T*-tests

### Physical activity and sedentary behavior

The accelerometer was worn 4–7 days (Fig. 2), on average close to 750 min per day (Table 3). Most subjects with less than 7 valid days forgot to wear the accelerometer at one or more full days. Others attached it too late or removed it too early during the day, resulting in less than 480 min of monitoring.

**Table 3:** Descriptors for daily physical activity and sedentary behavior

Parameter ^a^ (min∙day^− 1^)	Boys (*n* = 12)			Girls (*n* = 8)			*p*-value ^b^ (Boys vs Girls)	
	Mean	SD	Range	Mean	SD	Range		
Wear time	756	69	649–897	741	64	676–841	.6302	
Total PA	317	54	247–412	284	19	255–310	.1165	
- Light PA	255	39	201–323	251	16	229–279	.7878	
- Moderate PA	41	13	22–63	24	7	15–35	.0034	*
- Vigorous PA	22	10	4–40	9	4	5–18	.0027	*
MVPA	63	23	28–99	33	10	24–53	.0028	*
SB	439	92	345–651	457	66	389–552	.6399	

Abbreviations: *PA* physical activity, *MVPA* moderate-to-vigorous physical activity, *SB* sedentary behavior, vs versus. ^*^p < .005. ^a^ Obtained from accelerometry with epoch length 15 s (minutes per day: mean ± SD, range). ^**b**^ independent samples *T*-tests

**Fig. 3:**
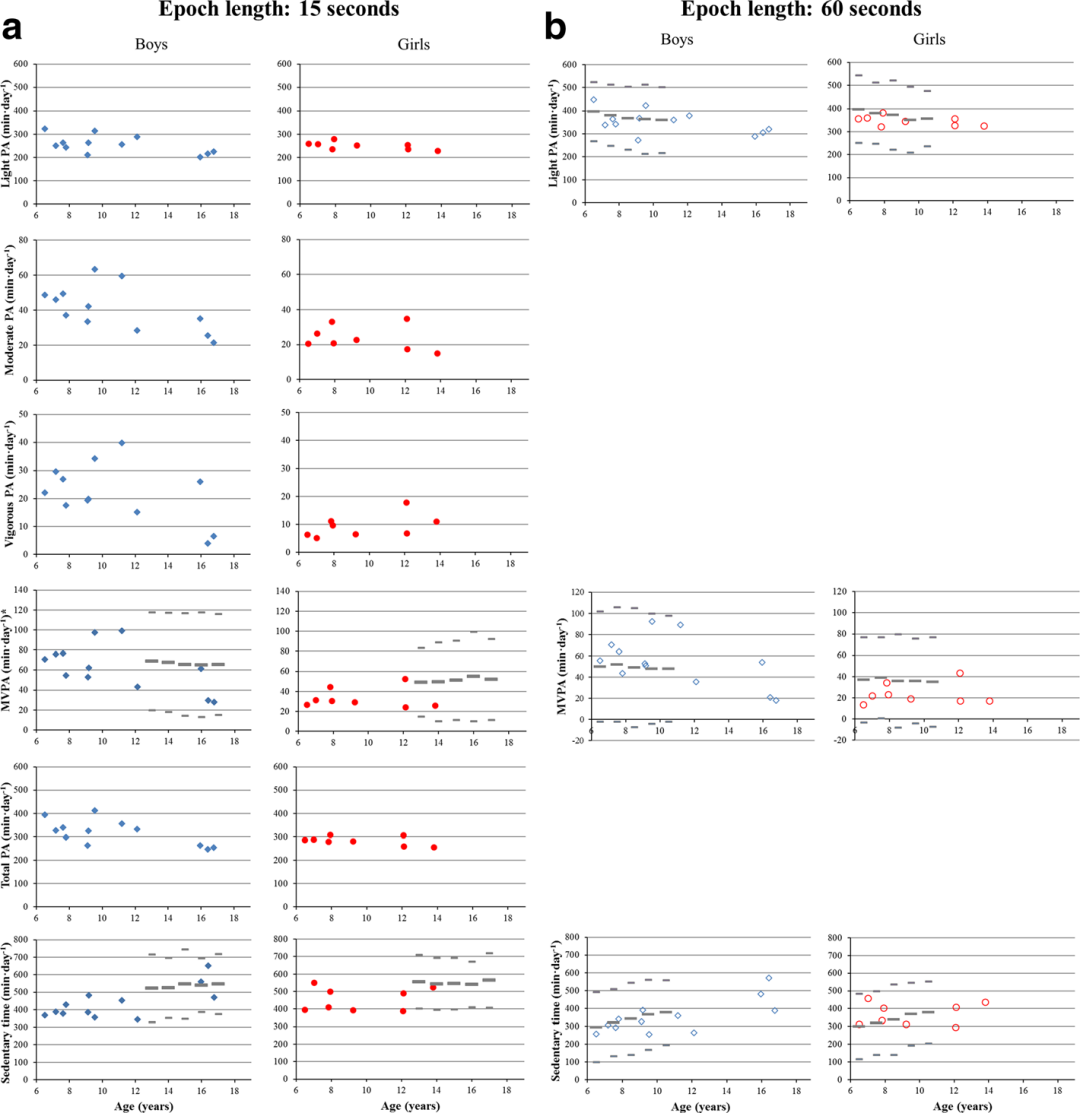
**a** and **b** Individual levels of daily physical activity and sedentary behavior, compared to non-burned peers. Time spent in various physical activity intensities and sedentary behavior, relative to age for both boys (left) and girls (right) after burn injury, calculated with an epoch length of (**a**) 15 s and (**b**) 60 s. Non-burned reference values (mean ± 2 SD) for adolescents aged 12.5–17.5 years are presented in (**a**) [20], and reference values for children aged 6–11 years are presented in (**b**) [21], because of the differences in epoch length. *Please note that the cut point for moderate-to-vigorous physical activity in the study of Ruiz et al. [20] was 2000 counts per minute (cpm) compared to the 2296 cpm of Evenson et al. [27] which was used in the current study. Abbreviations: PA = physical activity; MVPA = moderate-to-vigorous physical activity; min = minutes

Approximately 40% of the daily wear time was classified as physical activity (5.1 ± 0.8 h) and 60% was classified as sedentary behavior (7.4 ± 1.4 h) (Table 3). Most of the active time by far, 80% in boys and 88% in girls, was categorized as light physical activity and less than 10% as vigorous physical activity (7% in boys and 3% in girls). Although with large interindividual differences (Fig. 3), boys spent more time in moderate-to-vigorous physical activity than girls (*p* < .005, Table 3).

Exploratory linear regression analyses indicated that age was significantly associated with time spent in total physical activity. According to the regression line, time spent in total physical activity decreased by 6.7 min when age increased with 1 year (β − 6.7; 95% CI: -12.7 − − 0.7; *p* = .031). Time spent in sedentary behavior increased with 13.1 min by each year of age (β 13.1; 95% CI: 3.0–23.2; *p* = .014) (Fig. 3). Age explained 23.4% of the variance in time spent in total physical activity. The SEE was 41.6 (model *p* < .04) which indicates small error, given the range of 247–412 min of time spent in total physical activity (Table 3). In sedentary behavior, age explained 29.2% of the variance (SEE = 70.1, model *p* < .02). For time spent in moderate-to-vigorous physical activity, only sex was a significant predictor, explaining 39% of the variance (SEE = 19.0, model *p* < .01). The girls spent on average 29.5 min less time in moderate-to-vigorous physical activity than the boys in this study (β − 29.5; 95% CI: -47.7 − − 11.2; *p* = .003). None of the burn characteristics (%total body surface area burned, full thickness burns, leg involvement), time post burn, or length of hospital stay, were predictive for time spent in total physical activity, moderate-to-vigorous physical activity, or sedentary behavior.

**Fig. 4:**
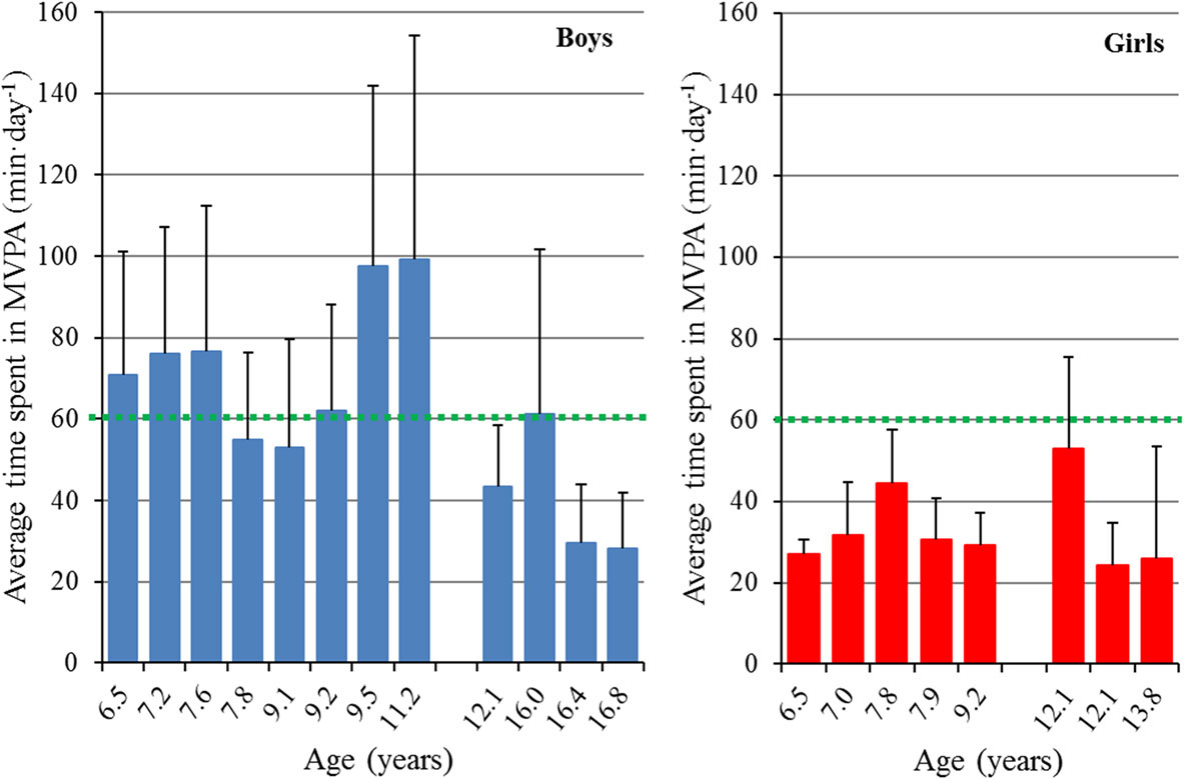
Attained versus recommended levels of moderate-to-vigorous physical activity (MVPA). Average daily time (mean ± SD) spent in MVPA, relative to age (child, adolescent), for both boys (left) and girls (right) after burn injury, compared to the World Health Organization physical activity recommendation of ≥60 min of MVPA per day (dotted line) [25]. Epoch length: 15 s

Comparison of individual scores with the WHO physical activity recommendation (Table 1) showed that 6 of the 8 boys aged < 12 years and 1 of the 4 boys aged ≥12 years achieved the recommended 60 min of daily moderate-to-vigorous physical activity (Fig. 4). None of the girls spent on average ≥ 60 min on moderate-to-vigorous physical activity per day.

### Comparison with non-burned peers

Reference values were only available for light physical activity, moderate-to-vigorous physical activity and sedentary behavior in children aged 6–11 years, and for moderate-to-vigorous physical activity and sedentary behavior in adolescents aged 12.5–17.5 years. On time spent in moderate-to-vigorous physical activity, most girls (6 out of 8) and adolescent boys (3 out of 4) scored below average, while most young boys (7 out of 8) scored on or above average. None of them, however, scored more than two SD from the non-burned reference mean (Fig. 3). Sedentary time of both boys and girls after burn injury was comparable with non-burned peers (Fig. 3), which was also true for time spent in light physical activity (Fig. 3b).

## Discussion

This preliminary investigation was the first study that objectively assessed intensity and duration of physical activity and sedentary behavior following pediatric burns and compared those with non-burned reference values. Accelerometer data showed that children and adolescents 1–5 years after moderate to severe burns were physically active about 5.1 h and sedentary for 7.4 h per day. The boys, although with large interindividual differences, spent more time on moderate and vigorous physical activity than the girls. Similar to the findings in non-burned pediatric populations [20, 21, 23, 24], older age was associated with less time spent in physical activity, while more time was spent in sedentary behavior. No trends were found indicating an effect of burn characteristics, time post burn, or length of hospital stay, although this might be a consequence of the cross-sectional design of this study and the limited sample size. No differences were found with non-burned reference values.

Time spent in moderate-to-vigorous physical activity in children and adolescents 1–5 years after burn injury was similar to that of non-burned peers. This is consistent with the results of Disseldorp et al. [17], who indicated that both physical fitness and muscle strength were not significantly different from non-burned peers in this population. In contrast, only 35% of the group, especially the boys aged < 12 years, spent on average ≥ 60 min of moderate-to-vigorous physical activity per day, which, according to the WHO, is crucial to reduce the risk of cardiovascular and metabolic diseases throughout life [25]. Seven subjects did not reach 60 min of moderate-to-vigorous physical activity on any of the monitoring days. In non-burned references, the percentage of children achieving the recommended amount of daily moderate-to-vigorous physical activity is low as well, ranging from 2.0 to 56.8% [20, 21]. These findings suggest that long-term health and well-being might be at risk in both groups. However, pediatric burn patients already are at increased risk of several physical and physiological diseases, e.g. pulmonary, musculoskeletal, and cardiovascular diseases, in the long term [10–13]. Adequate levels of physical activity generally can help to prevent the development of such conditions [1] and this might also be true for pediatric burn patients. Therefore, it is deemed important to encourage physical activity and sports in this vulnerable population.

It is important to note, however, that there is no consensus yet on the best cut points for the classification of moderate-to-vigorous physical activity in children and adolescents [26]. According to the cut points of Evenson et al. [27], which are recommended for children and youth [28] and therefore applied in the current study, intensities of > 2296 counts per minute (cpm) are considered moderate-to-vigorous physical activity. In the literature, however, thresholds ranging from 1000 to 4000 have been used. The WHO states that an intensity of ≥3 MET (metabolic equivalent) is considered moderate-to-vigorous physical activity, however it is unclear which accelerometer counts are associated with that intensity. The height of this threshold of course has major consequences for the total number of subjects that is classified as meeting the WHO physical activity recommendation (Table 1). If our threshold for moderate-to-vigorous physical activity is higher than the threshold intended by the WHO, this would explain the low proportion of subjects that achieved the recommended amount of daily moderate-to-vigorous physical activity. When it comes to patients, it is also important to realize that the cut points recommended for healthy children might result in misclassification of activity intensity in children with a chronic condition [29]. Physiological or biomechanical limitations might require them to work at a higher energy level to complete the same task and thus reach similar accelerometer counts [29–31]. Therefore, before generalized conclusions can be made regarding the appropriateness of physical activity levels after pediatric burns, consensus is needed concerning the best cut point used to classify moderate-to-vigorous physical activity, for both healthy children and children with a (chronic) condition.

Most of the evidence linking sedentary behavior to health outcomes in children and adolescents has focused on screen time [3]. Watching television for more than 2 hours per day has been associated with negative health outcomes like obesity, decreased physical fitness, increased blood pressure, lower self-esteem, social behavioral problems, and decreased academic achievement in school-aged children and youth (5–17 years) [3, 32]. As accelerometry does not distinguish between screen time and other types of sedentary behavior (school, motorized transport, leisure time), it was not possible to determine whether our pediatric burn patients spent too much time watching television or playing computer games. Surprisingly, the current literature provides limited evidence for adverse effects of total time spent in sedentary behavior on health and well-being in children and adolescents [32]. Nevertheless, given the adverse effects among adults, and some evidence of tracking of sedentary behavior across the life course, encouraging children and adolescents to limit their time spent in sedentary behavior for now seems prudent [32].

Although body composition and body weight might also influence physical activity and sedentary behavior, we chose not to include these variables in our regression analyses for several reasons. First, the relationship between body weight / body composition and physical activity and sedentary behavior is probably mutual. Overweight can affect physical activity and encourage sedentary behavior, but, on the other hand, overweight can be the result of poor activity levels and abundant sedentary time. Second, both body weight and body composition are directly related to age and sex in pediatric populations. Healthy body mass index (BMI) increases with age and is different for boys and girls. Adding body weight or BMI to the regression model could therefore lead to over adjustment and create an apparent effect when none exists [33]. We did assess whether our subjects were classified, with regard to age and sex, as being overweight, underweight, or having healthy weight. All subjects were classified as having healthy weight, except for two girls who were classified as being severely overweight, and one girl was classified as being underweight. Activity levels and sedentary behavior of those three subjects did not deviate from the others, so it was concluded that further analysis would not be of additional value.

Accelerometry is one of the most valid methods to gain insight in habitual activity levels [34, 35], and has frequently been used to assess time spent in physical activity and sedentary behavior in children and adolescents, also in special populations [36–43]. However, comparability among studies is limited, due to the variety in epoch lengths, cut points, and outcome variables. For accurate comparison, accelerometer data should be processed and summarized similarly and in a transparent manner. For those reasons, we chose to adopt the decisions in data reduction and analysis from our reference studies, and to apply the algorithm of Heil et al. [22] for transparency of our methods. To compare our patients data with the results of Konstabel et al. [21], we needed to analyze our accelerometer data with the less favorable epoch length of 60 s, resulting in significantly different outcomes compared to the 15 s–epoch analysis [see Additional file 1]. Time spent in total physical activity and light physical activity calculated from 60s–epochs was significantly higher (*p* < .0001) compared to similar calculations based on 15 s–epochs. Time spent in both vigorous physical activity (and consequently moderate-to-vigorous physical activity) and sedentary behavior, on the other hand, was significantly lower (*p* < .0001) when 60s–epochs were used [see Additional file 1]. Individual level analyses showed that this was a systematic effect of the data processing method [see Additional file 2]. As the physical activity patterns of children and adolescents are typically characterized by frequent, short duration bursts of vigorous physical activity, this effect of epoch length is understandable. Using a 60 s epoch, short bursts of vigorous physical activity will be averaged over the minute and remain undetected. A minute of sedentary behavior, on the other hand, can be incorrectly classified as light physical activity when it contains only one short burst of vigorous physical activity [44, 45]. To obtain a ‘real’ picture of physical activity and sedentary behavior in children and adolescents, short epoch lengths are thus essential. A study by Edwardson and Gorely [45] suggests that a 5 s epoch would be most appropriate to detect the typical short bursts of vigorous physical activity in children and adolescents. Bland-Altman plots showed however reasonable agreement between the results obtained with 5 and 15 s epochs, which suggests that the results of studies using those epoch lengths could be compared. Studies using 60s–epochs, on the other hand, should not be compared with studies applying epochs ≤15 s [45]. This implies that new accelerometry reference values are needed for children aged 6–11 years, obtained with an epoch length of ≤15 s.

Some limitations of this study need to be discussed. First of all, participation in this study was on a voluntary basis. It is conceivable that this resulted in selection bias. It is unclear, however, how this has affected our results. It could be that those who were already interested or involved in regular physical activity were most willing to participate. On the other hand, those who still experienced restrictions with activities and participation may have been eager to volunteer for this study.

Secondly, the cross-sectional design of this study and the small number of subjects, make it difficult to explain variance with multiple regression modeling. Nevertheless, the decline in time spent in physical activity and increase in time in sedentary behavior with age in children and adolescents observed in this study is a well-known phenomenon that is also reported in larger, longitudinal studies [23, 24]. As physical activity is known to be affected by social environment, i.e. socioeconomic state, it would have also been interesting to find out whether socioeconomic state could explain some variance in physical activity or sedentary behavior in our study population. Unfortunately, we did not obtain this information from our subjects and could therefore not control for this factor in our analyses.

This preliminary investigation is a first and important start to gain insight in daily time spent in physical activity and sedentary behavior in children and adolescents following moderate-to-severe burns. However, further research is required to obtain a full picture of post burn physical activity levels and to identify those patients who are at greatest risk of inactivity. It would be interesting to examine activity patterns in children who are closer to their burn and to assess how our subjects will do in several years, when they become adults. It has been shown that physical fitness is low in a large proportion of adult patients even decades post-burn [46]. It would therefore be interesting to examine activity patterns of adult burn patients as well.

## Conclusions

Duration and intensity of physical activity and sedentary behavior in children and adolescents 1–5 years after burn injury were similar to non-burned peers. However, only 35% of the group met the WHO physical activity recommendation. Given the increased risk for physical conditions following pediatric burns, physical activity should be encouraged in this vulnerable population.

## Additional files

Additional file 1: Accelerometry outcomes calculated from 15 s–epochs and 60s–epochs. As the physical activity patterns of children are typically characterized by frequent, short duration bursts of vigorous physical activity, short epoch lengths are essential to obtain a ‘real’ picture of their physical activity and sedentary behavior. The significant differences in accelerometry outcomes calculated from 15 s–epochs and 60s–epochs indicate that the use of 60s–epochs should be discouraged in pediatric populations. (DOCX 28 kb)

Additional file 2: The systematic effect of epoch length. This figure shows the systematic overestimation of time spent in light physical activity and the systematic underestimation of both time in vigorous physical activity and sedentary behavior, when 60s–epochs are used rather than 15 s–epochs. Abbreviations: SB = sedentary behavior; PA = physical activity; min = minutes, sec = second. (TIFF 969 kb)

## Abbreviations

SD: Standard deviation
SEE: Standard error of the estimate
WHO: World Health Organization

## References

[1] Janssen I, Leblanc AG (2010). Systematic review of the health benefits of physical activity and fitness in school-aged children and youth. Int J Behav Nutr Phys Act.

[2] Biddle SJ, Asare M (2011). Physical activity and mental health in children and adolescents: a review of reviews. Br J Sports Med.

[3] de Rezende LF, Rodrigues Lopes M, Rey-Lopez JP, Matsudo VK, Luiz Odo C (2014). Sedentary behavior and health outcomes: an overview of systematic reviews. PLoS One.

[4] Maggio AB, Hofer MF, Martin XE, Marchand LM, Beghetti M, Farpour-Lambert NJ (2010). Reduced physical activity level and cardiorespiratory fitness in children with chronic diseases. Eur J Pediatr.

[5] Gore DC, Chinkes DL, Wolf SE, Sanford AP, Herndon DN, Wolfe RR (2006). Quantification of protein metabolism in vivo for skin, wound, and muscle in severe burn patients. JPEN J Parenter Enteral Nutr.

[6] Jeschke MG, Gauglitz GG, Kulp GA, Finnerty CC, Williams FN, Kraft R, Suman OE, Mlcak RP, Herndon DN (2011). Long-term persistance of the pathophysiologic response to severe burn injury. PLoS One.

[7] Porter C, Herndon DN, Sidossis LS, Borsheim E (2013). The impact of severe burns on skeletal muscle mitochondrial function. Burns.

[8] Akkerman M, Mouton LJ, Dijkstra F, Niemeijer AS, van Brussel M, van der Woude LHV, Disseldorp LM, Nieuwenhuis MK. Perceived fatigue following pediatric burns. Burns. 2017; 10.1016/j.burns.2017.05.007. 10.1016/j.burns.2017.05.007 28610795

[9] Grice KO, Barnes KJ, Vogel KA (2015). Influence of burn injury on activity participation of children. J Burn Care Res.

[10] Stone J, Gawaziuk JP, Khan S, Chateau D, Bolton JM, Sareen J, Enns J, Doupe M, Brownell M, Logsetty S (2016). Outcomes in adult survivors of childhood burn injuries as compared with matched controls. J Burn Care Res.

[11] Duke JM, Randall SM, Fear MW, Boyd JH, Rea S, Wood FM. Respiratory morbidity after childhood burns: a 10-year follow-up study. Pediatrics. 2016;138 10.1542/peds.2016-1658. 10.1542/peds.2016-1658 27664086

[12] Duke JM, Randall SM, Fear MW, Boyd JH, Rea S, Wood FM (2015). Long-term effects of pediatric burns on the circulatory system. Pediatrics.

[13] Duke JM, Randall SM, Fear MW, Boyd JH, Rea S, Wood FM (2015). Increased admissions for musculoskeletal diseases after burns sustained during childhood and adolescence. Burns.

[14] Duke JM, Rea S, Boyd JH, Randall SM, Wood FM (2015). Mortality after burn injury in children: a 33-year population-based study. Pediatrics.

[15] Suman OE, Spies RJ, Celis MM, Mlcak RP, Herndon DN (2001). Effects of a 12-wk resistance exercise program on skeletal muscle strength in children with burn injuries. J Appl Physiol.

[16] Alloju SM, Herndon DN, McEntire SJ, Suman OE (2008). Assessment of muscle function in severely burned children. Burns.

[17] Disseldorp LM, Mouton LJ, Van der Woude LH, Van Brussel M, Nieuwenhuis MK (2015). Anthropometry, muscular strength and aerobic capacity up to 5 years after pediatric burns. Burns.

[18] Disseldorp LM, Mouton LJ, Takken T, Van Brussel M, Beerthuizen GI, Van der Woude LH, Nieuwenhuis MK (2012). Design of a cross-sectional study on physical fitness and physical activity in children and adolescents after burn injury. BMC Pediatr.

[19] Hanggi JM, Phillips LR, Rowlands AV (2013). Validation of the GT3X ActiGraph in children and comparison with the GT1M ActiGraph. J Sci Med Sport.

[20] Ruiz JR, Ortega FB, Martinez-Gomez D, Labayen I, Moreno LA, De Bourdeaudhuij I, Manios Y, Gonzalez-Gross M, Mauro B, Molnar D (2011). Objectively measured physical activity and sedentary time in European adolescents: the HELENA study. Am J Epidemiol.

[21] Konstabel K, Veidebaum T, Verbestel V, Moreno LA, Bammann K, Tornaritis M, Eiben G, Molnar D, Siani A, Sprengeler O (2014). Objectively measured physical activity in European children: the IDEFICS study. Int J Obes.

[22] Heil DP, Brage S, Rothney MP (2012). Modeling physical activity outcomes from wearable monitors. Med Sci Sports Exerc.

[23] Corder K, Sharp SJ, Atkin AJ, Griffin SJ, Jones AP, Ekelund U, van Sluijs EM (2015). Change in objectively measured physical activity during the transition to adolescence. Br J Sports Med.

[24] Nelson MC, Neumark-Stzainer D, Hannan PJ, Sirard JR, Story M (2006). Longitudinal and secular trends in physical activity and sedentary behavior during adolescence. Pediatrics.

[25] World Health Organization. Factsheet physical activity and young people: Recommended levels of physical activity for children aged 5–17 years. http://www.who.int/dietphysicalactivity/factsheet_young_people/en/. Accessed 4 May 2016.

[26] Kim Y, Beets MW, Welk GJ (2012). Everything you wanted to know about selecting the “right” Actigraph accelerometer cut-points for youth, but...: a systematic review. J Sci Med Sport.

[27] Evenson KR, Catellier DJ, Gill K, Ondrak KS, McMurray RG (2008). Calibration of two objective measures of physical activity for children. J Sports Sci.

[28] Trost SG, Loprinzi PD, Moore R, Pfeiffer KA (2011). Comparison of accelerometer cut points for predicting activity intensity in youth. Med Sci Sports Exerc.

[29] Stephens S, Takken T, Esliger DW, Pullenayegum E, Beyene J, Tremblay M, Schneiderman J, Biggar D, Longmuir P, McCrindle B (2016). Validation of accelerometer prediction equations in children with chronic disease. Pediatr Exerc Sci.

[30] Shakur YA, Richards H, Pencharz PB (2008). Is it necessary to measure resting energy expenditure in clinical practice in children?. J Pediatr.

[31] Durstine JL, Painter P, Franklin BA, Morgan D, Pitetti KH, Roberts SO (2000). Physical activity for the chronically ill and disabled. Sports Med.

[32] Cliff DP, Hesketh KD, Vella SA, Hinkley T, Tsiros MD, Ridgers ND, Carver A, Veitch J, Parrish AM, Hardy LL (2016). Objectively measured sedentary behaviour and health and development in children and adolescents: systematic review and meta-analysis. Obes Rev.

[33] Schisterman EF, Cole SR, Platt RW (2009). Overadjustment bias and unnecessary adjustment in epidemiologic studies. Epidemiology.

[34] Westerterp KR (2009). Assessment of physical activity: a critical appraisal. Eur J Appl Physiol.

[35] Lubans DR, Hesketh K, Cliff DP, Barnett LM, Salmon J, Dollman J, Morgan PJ, Hills AP, Hardy LL (2011). A systematic review of the validity and reliability of sedentary behaviour measures used with children and adolescents. Obes Rev.

[36] Braam KI, van Dijk-Lokkart EM, Kaspers GJ, Takken T, Huisman J, Bierings MB, Merks JH, van de Heuvel-Eibrink MM, van Dulmen-den Broeder E, Veening MA (2016). Cardiorespiratory fitness and physical activity in children with cancer. Support Care Cancer.

[37] Henderson CJ, Lovell DJ, Specker BL, Campaigne BN (1995). Physical activity in children with juvenile rheumatoid arthritis: quantification and evaluation. Arthritis Care Res.

[38] Heath JA, Ramzy JM, Donath SM (2010). Physical activity in survivors of childhood acute lymphoblastic leukaemia. J Paediatr Child Health.

[39] Aznar S, Gallardo C, Fiuza-Luces C, Santana-Sosa E, Lopez-Mojares LM, Santalla A, Rodriguez-Romo G, Perez M, Garatachea N, Lucia A (2014). Levels of moderate--vigorous physical activity are low in Spanish children with cystic fibrosis: a comparison with healthy controls. J Cyst Fibros.

[40] Norgaard M, Twilt M, Andersen LB, Herlin T (2016). Accelerometry-based monitoring of daily physical activity in children with juvenile idiopathic arthritis. Scand J Rheumatol.

[41] Kashikar-Zuck S, Flowers SR, Verkamp E, Ting TV, Lynch-Jordan AM, Graham TB, Passo M, Schikler KN, Hashkes PJ, Spalding S (2010). Actigraphy-based physical activity monitoring in adolescents with juvenile primary fibromyalgia syndrome. J Pain.

[42] Gonzalez LM, Peiro-Velert C, Devis-Devis J, Valencia-Peris A, Perez-Gimeno E, Perez-Alenda S, Querol F (2011). Comparison of physical activity and sedentary behaviours between young haemophilia a patients and healthy adolescents. Haemophilia.

[43] Ryan JM, Forde C, Hussey JM, Gormley J (2015). Comparison of patterns of physical activity and sedentary behavior between children with cerebral palsy and children with typical development. Phys Ther.

[44] Nettlefold L, Naylor PJ, Warburton DE, Bredin SS, Race D, McKay HA (2016). The influence of epoch length on physical activity patterns varies by Child's activity level. Res Q Exerc Sport.

[45] Edwardson CL, Gorely T (2010). Epoch length and its effect on physical activity intensity. Med Sci Sports Exerc.

[46] Ganio MS, Pearson J, Schlader ZJ, Brothers RM, Lucas RA, Rivas E (2015). Aerobic Fitness is Disproportionately low in adult Burn Survivors Years After Injury. Journal of burn care & research: official publication of the American Burn Association.

[47] Sedentary Behaviour Research N (2012). Letter to the editor: standardized use of the terms “sedentary” and “sedentary behaviours”. Appl Physiol Nutr Metab.

[48] Tremblay MS, LeBlanc AG, Kho ME, Saunders TJ, Larouche R, Colley RC, Goldfield G, Connor Gorber S (2011). Systematic review of sedentary behaviour and health indicators in school-aged children and youth. Int J Behav Nutr Phys Act.

[49] Australian Government Department of Health. Australia’s Physical Activity and Sedentary Behaviour Guidelines for Children (5–12 years) and Young People (13–17 years). http://www.health.gov.au/internet/main/publishing.nsf/Content/health-pubhlth-strateg-phys-act-guidelines. Accessed 4 May 2016.

