# Supplementary material for: Physical activity and sedentary behavior following pediatric burns – a preliminary investigation using objective activity monitoring
Source: BMC Sports Sci Med Rehabil. 2018 Feb 9;10:4. doi: 10.1186/s13102-018-0093-5 (PMC5807851; doi:10.1186/s13102-018-0093-5)
Supplement: Supplementary file 1 — Accelerometry outcomes calculated from 15 s–epochs and 60s–epochs. As the physical activity patterns of children are typically characterized by frequent, short duration bursts of vigorous physical activity, short epoch lengths are essential to obtain a ‘real’ picture of their physical activity and sedentary behavior. The significant differences in accelerometry outcomes calculated from 15 s–epochs and 60s–epochs indicate that the use of 60s–epochs should be discouraged in pediatric populations. (DOCX 28 kb) [file 13102_2018_93_MOESM1_ESM.docx]

**Additional file 1 Accelerometry outcomes calculated from 15s-epochs and 60s-epochs.**

| **Parameter**  **(min∙day^-1^)** |  | **15 seconds epoch** | |  | **60 seconds epoch** | |  | ***p*-value**  **(15s vs 60s)** | |
| --- | --- | --- | --- | --- | --- | --- | --- | --- | --- |
|  |  | **mean** | **SD** |  | **mean** | **SD** |  |  |  |
| Total PA |  | 304 | 46 |  | 391 | 54 |  | <.0001 | * |
|  |  |  |  |  |  |  |  |  |  |
| Light PA |  | 253 | 31 |  | 350 | 41 |  | <.0001 | * |
| Moderate PA |  | 34 | 14 |  | 32 | 18 |  | .07 |  |
| Vigorous PA |  | 17 | 10 |  | 10 | 7 |  | <.0001 | * |
|  |  |  |  |  |  |  |  |  |  |
| MVPA |  | 51 | 24 |  | 42 | 24 |  | <.0001 | * |
|  |  |  |  |  |  |  |  |  |  |
| SB |  | 446 | 81 |  | 360 | 83 |  | <.0001 | * |

Abbreviations: s=seconds; min=minutes; PA=physical activity; MVPA=moderate-to-vigorous physical activity; SB=sedentary behavior; vs=versus. ^*^*p*<.0001
