# Supplementary figures and images for: Physical activity and sedentary behavior following pediatric burns – a preliminary investigation using objective activity monitoring
Source: BMC Sports Sci Med Rehabil. 2018 Feb 9;10:4. doi: 10.1186/s13102-018-0093-5 (PMC5807851; doi:10.1186/s13102-018-0093-5)

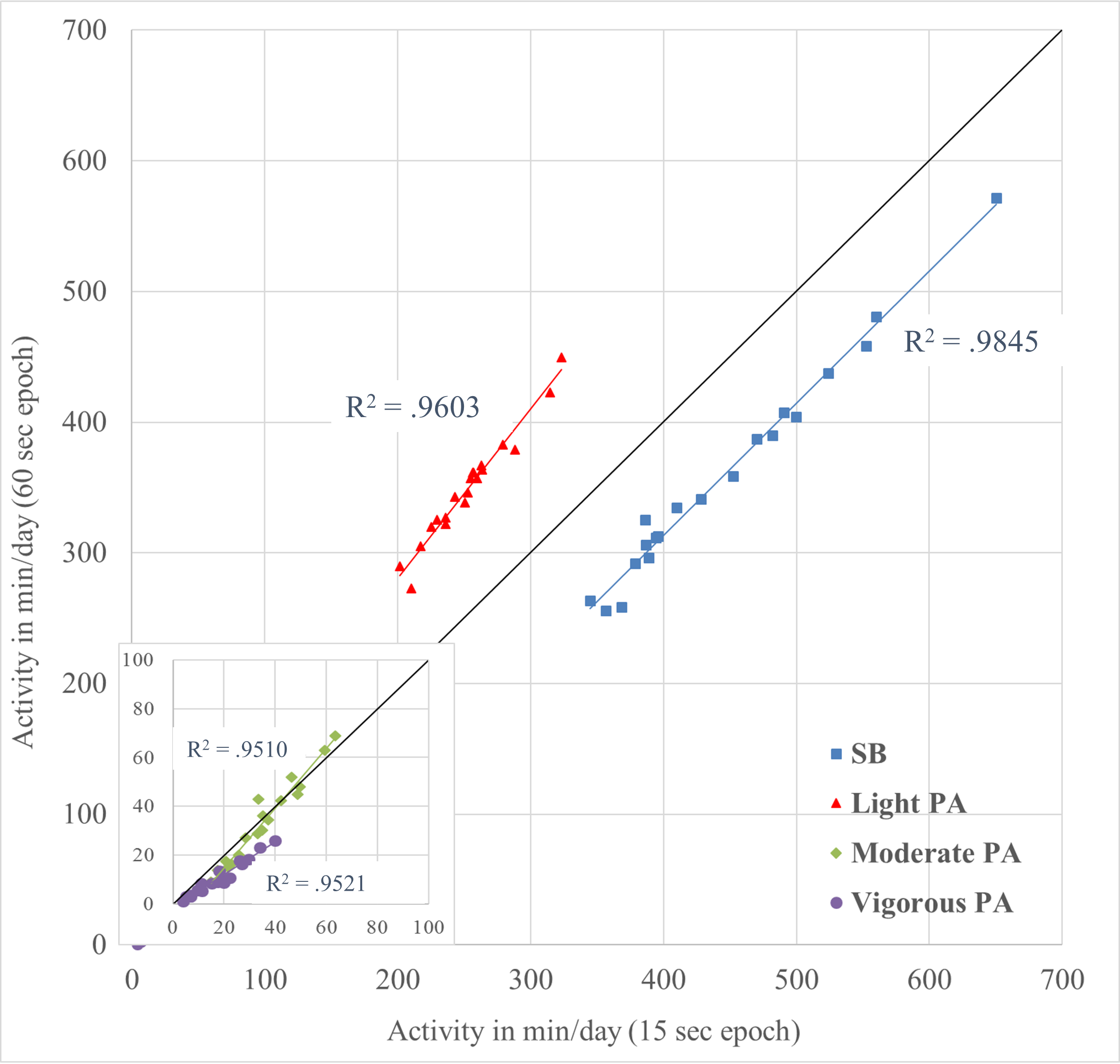

Supplement: Supplementary file 2 — The systematic effect of epoch length. This figure shows the systematic overestimation of time spent in light physical activity and the systematic underestimation of both time in vigorous physical activity and sedentary behavior, when 60s–epochs are used rather than 15 s–epochs. Abbreviations: SB = sedentary behavior; PA = physical activity; min = minutes, sec = second. (TIFF 969 kb) [file 13102_2018_93_MOESM2_ESM.tif]
